# Metatranscriptomic analysis shows functional alterations in subgingival biofilm in young smokers with periodontitis: a pilot study

**DOI:** 10.1590/1678-7757-2024-0031

**Published:** 2024-08-16

**Authors:** Renato Corrêa Viana CASARIN, Rafaela Videira Clima da Silva, Hélvis Enri de Sousa PAZ, Camila Schmidt STOLF, Lucas Miguel CARVALHO, Melline Fontes NORONHA, Antonio Wilson SALLUM, Mabelle de Freitas MONTEIRO

**Affiliations:** 1 Universidade Estadual de Campinas Faculdade de Odontologia de Piracicaba Departamento de Prótese e Periodontia Piracicaba Brasil Universidade Estadual de Campinas, Faculdade de Odontologia de Piracicaba, Departamento de Prótese e Periodontia, Piracicaba, Brasil.; 2 Universidade Estadual de Campinas Centro de Pesquisas em Engenharias e Ciências Computacionais Campinas Brasil Universidade Estadual de Campinas, Centro de Pesquisas em Engenharias e Ciências Computacionais, Campinas, Brasil.; 3 University of Illinois at Chicago Research Resource Center Research Informatics Core Illinois USA University of Illinois at Chicago, Research Resource Center, Research Informatics Core, Illinois, USA.

**Keywords:** Smoking, Periodontitis, RNA-Seq, Host-pathogen interactions, Gene expression, Oral microbiology, Non-invasive diagnostics

## Abstract

This study aimed to assess the influence of smoking on the subgingival metatranscriptomic profile of young patients affected by stage III/IV and generalized periodontal disease.

**Methodology:**

In total, six young patients, both smokers and non-smokers (n=3/group), who were affected by periodontitis were chosen. The STROBE (Strengthening the Reporting of Observational Studies in Epidemiology) guidelines for case-control reporting were followed. Periodontal clinical measurements and subgingival biofilm samples were collected. RNA was extracted from the biofilm and sequenced via Illumina HiSeq. Differential expression analysis used Kyoto Encyclopedia of Genes and Genomes (KEGG) enrichment, and differentially expressed genes were identified using the Sleuth package in R, with a statistical cutoff of ≤0.05.

**Results:**

This study found 3351 KEGGs in the subgingival biofilm of both groups. Smoking habits altered the functional behavior of subgingival biofilm, resulting in 304 differentially expressed KEGGs between groups. Moreover, seven pathways were modulated: glycan degradation, galactose metabolism, glycosaminoglycan degradation, oxidative phosphorylation, peptidoglycan biosynthesis, butanoate metabolism, and glycosphingolipid biosynthesis. Smoking also altered antibiotic resistance gene levels in subgingival biofilm by significantly overexpressing genes related to beta-lactamase, permeability, antibiotic efflux pumps, and antibiotic-resistant synthetases.

**Conclusion:**

Due to the limitations of a small sample size, our data suggest that smoking may influence the functional behavior of subgingival biofilm, modifying pathways that negatively impact the behavior of subgingival biofilm, which may lead to a more virulent community.

## Graphical abstract

**Figure:**
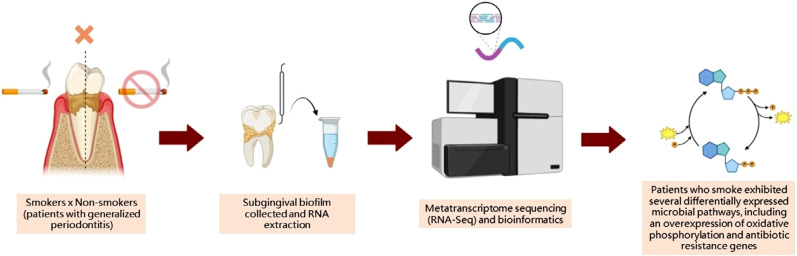

## Introduction

Smoking is a widely recognized major risk factor for the progression and severity of periodontal diseases as it is associated with increased probing depth, clinical attachment loss, gingival recession, and a higher likelihood of future tooth loss than in non-smokers. Additionally, periodontal patients experience worse clinical outcomes after periodontal therapy.^1^ Tobacco consumption is widespread among young adults, often beginning in adolescence.^2^ Consequently, individuals who develop periodontal diseases at a young age may experience a poorer association much earlier in life.

Clinical evidence shows that the negative impact of smoking is even more pronounced at early ages. Studies associating smoking with aggressive periodontitis indicate that these patients show a significant number of non-responsive sites to non-surgical therapy, with a higher risk of long-term disease recurrence.^3^ Moreover, even after periodontal treatment, these patients seem to show a faster subgingival recolonization by periodontopathogens than non-smokers,^4^ suggesting that smoking may locally modify the subgingival community in parallel with dysfunctional immune responses and dysbiosis.

The microbial community of smoking-associated periodontitis is taxonomically less diverse and distinct than that in non-smokers, and periodontally healthy smokers show a subgingival microbiome composition closely related to that in diseased subjects.^5^ The expression of virulence factors or of other genes favoring host stimulation could be more impacting than taxonomy since commensal bacteria — rather than only well-known pathogens — can modify community behavior and impact functional microbial content. An *in vitro* study showed that smoke exposure was associated with transcriptional shifts in biofilm, increasing virulence gene expression and creating an anaerobic, proinflammatory, and pathogen-rich environment.^6^

Thus, metatranscriptomics has successfully characterized the functional signatures of the subgingival biofilm from healthy and diseased patients.^7^ However, the comparison between distinct diseased environments and the effect of heavy smoking habits on those environments is yet to be performed.

Therefore, this study aims to investigate whether the additional influence of smoking habits alters the gene expression profile of the subgingival biofilm in individuals suffering from generalized periodontal disease.

## Methodology

### Study design

The influence of smoking on the subgingival transcriptome of young subjects with generalized grade C periodontitis was evaluated in this cross-sectional study (formerly known as “generalized aggressive periodontitis”). This research was approved by the local ethics committee (088325/2017). Written informed consent was obtained from all participants.

### Study Population

Non-smokers (PerioCNsmk) and smokers (PerioCSmk) were chosen according to the following inclusion criteria: (1) diagnosis of generalized periodontitis Grade C Stage 3-4 (PerioC);^8^ (2) age below 35 years at the moment of diagnosis and systemic health evaluation; (3) presence of at least 15 teeth; (4) presence of at least six teeth with deep sites (≥7 mm) in areas other than bifurcations. Patients were considered smokers if they had consumed more than 10 cigarettes a day for at least five years, whereas non-smokers were those without a history of smoking. Sample size was determined considering the number of samples in previous preliminary studies employing RNAseq analysis.^9,10^ In total, six patients were included in this study, 3 in each group. The STROBE (Strengthening the Reporting of Observational Studies in Epidemiology) guidelines for case-control reporting were adopted in this study.^11^

The following were considered as exclusion criteria: (1) periapical or pulp alterations; (2) systemic alteration or use of medications that may influence response to periodontal treatment (such as antibiotics and anti-inflammatories) six months before this study; (3) pregnant and lactating women; (4) periodontal treatment (including subgingival instrumentation) in the six months preceding this study; (5) teeth with bifurcation involvement; (6) tooth mobility degree ≥2;^12^ (7) oral pathologies; (8) history of allergy to any component of this study, and (9) a history of periodontal surgery.

### Clinical measurements

The plaque index (PI - %),^13^ bleeding on probing index (BoP - %),^14^ probing depth (PD - mm), clinical attachment level (CAL - mm), and gingival recession (GR - mm) were measured at six sites per tooth (mesiobuccal, buccal, distobuccal, distolingual, lingual and mesiolingual) in all teeth excluding third molars. A calibrated standard probe (UNC-15, Hu-Friedy, Chicago, IL, USA) and the same calibrated clinician (RVCS) performed the measurements. Intra‐class correlation showed 91% reproducibility for CAL and 94% for PD. Calibration was conducted on three Grade C periodontitis subjects who had no involvement with this study. Each subject underwent two examinations in two sessions with a 24-hour interval between them.

### Biofilm collection and RNA extraction

Supragingival plaque was first removed, and the areas were thoroughly dried and isolated with cotton rolls. In total, six interproximal sites with the deepest periodontal probing depth (PD ≥ 5 mm) were chosen. Bifurcation areas and third molars were excluded. Subgingival biofilm samples were collected using periodontal curettes (Hu-Friedy, Chicago, IL, USA). After a single collection in each site, samples from the same patient were pooled together and immediately stored in Eppendorf tubes containing 100μl of RNA storage reagent (RNAlater™ Stabilization Solution, Thermo Fisher Scientific, MA, USA).

Total RNA was extracted by a specific kit (RNAeasy^®^ Mini Kit extraction, Valencia, CA, USA) after the following extraction buffers were added: Lysozyme 20 mg/ml (Thermo Fisher Scientific, MA, USA) + Mutanolysin 5,000U / ml (Thermo Fisher Scientific, MA, USA) + Tris- 1M HCl, pH 8.0. The extracted RNA was stored in a freezer at −80 °C until RNA sequencing.

#### Metatranscriptome sequencing

RNA was sequenced via the Illumina HiSeq 2,500 platform. RNA-seq libraries were prepared using a specific kit (TruSeq™ RNA Sample Prep Kit v2, Illumina, Inc.; San Diego CA, USA) according to the manufacturer’s instructions. The adjusted libraries were sequenced in the same lane with 2×100-bp paired-end reads on the Illumina HiSeq sequencer. The RNA-seq data obtained in this study are available in the Sequence Read Archive repository, https://www.ncbi.nlm.nih.gov/bioproject/PRJNA757462. Quality control was analyzed by the FastQC software.^15^ Trimmomatic v0.39^16^ was used to clear reads with a quality lower than Phred 20 (Q20). We extracted microorganism sequences based on two steps. In the first step, the reads aligned to the human genome (hg38) were eliminated by mapping with bowtie2.^17^ In the second step, based on these filtered data, SortMeRNA^18^ was run to filter the ribosomal RNA from the metatranscriptomic data. The reference microbiome was generated based on the Human Oral Microbiome Database (HOMD)^19^ and the de novo assembly of the unmapped reads in the HOMD. The cleaned reads were aligned in HOMD using bowtie2, and the de novo sequences from unaligned reads were assembled using Trinity v2.8.3.^20^ Data quantification at the gene and transcript level was performed on Kallisto v0.49^21^. Differentially expressed genes were identified using the Sleuth v0.30.0 package,^22^ considering a q-value cutoff <= 0.05. The functional orthologs, called the KO (KEGG Orthology) group, rather than a single gene or protein, were identified by the KEGG database^23^ to find experimental evidence in a specific organism that can be extended to other organisms in the microbiome. KO analysis used the complete list of identifiers in the enrichment pathway analysis to reconstruct the predicted pathways using the FMAP pipeline.^24^ Additionally, the sequences obtained after alignment with HOMD and de novo assembly step were screened and matched for transcripts related to antibiotic resistance, using a comprehensive antibiotic resistance database as reference.^25^

## Statistical analysis

Means and standard deviations were calculated for the clinical parameters. For clinical measurements, data were initially tested for normal distribution by the Shapiro-Wilk test. Numeric demographic parameters with normal distribution were analyzed by the non-paired t-test, and categorical frequency data were analyzed by the chi-squared test. All tests were performed with a 5% significance level on SIGMAplot (Systat Software Inc., United States).

## Results

### Demographic and clinical data of the study subjects

Table 1 summarizes the demographic and clinical characteristics of PerioC smokers and non-smokers. Participants’ age and gender showed no significant differences (p>0.05). No differences occurred between groups for each periodontal parameter (p>0.05), showing a similar degree of periodontal destruction between subjects. Moreover, this study found no differences in probing depth between collection sites (p>0.05).

**Table 1:** Demographic and clinical data for the PerioCSmk and PerioCNSmk groups.

	PerioCSmk (n=3)	PerioCNSmk (n=3)
Gender (%F)	67	67
Age (years)	34.33±1.52	36.33±2.52
PD (mm±SD)	3.58±0.30	3.69±1.78
PI (%±SD)	48.26±14.86	51.00±0.50
BoP (%±SD)	48.93±44.20	54.00±0.50
CAL (mm±SD)	3.72±0.13	3.77±1.81
PD collected sites (mm±SD)	7.00±1.00	6.29±0.95

No differences in parameters occurred between PerioCSmk and PerioCNSmk. (Student’s t-test, considering p<0.05). PI: Plaque index; BoP: bleeding on probing index; PD: probing depth; CAL: clinical attachment level.

### Differentially expressed genes for smokers

**Figure 1:**
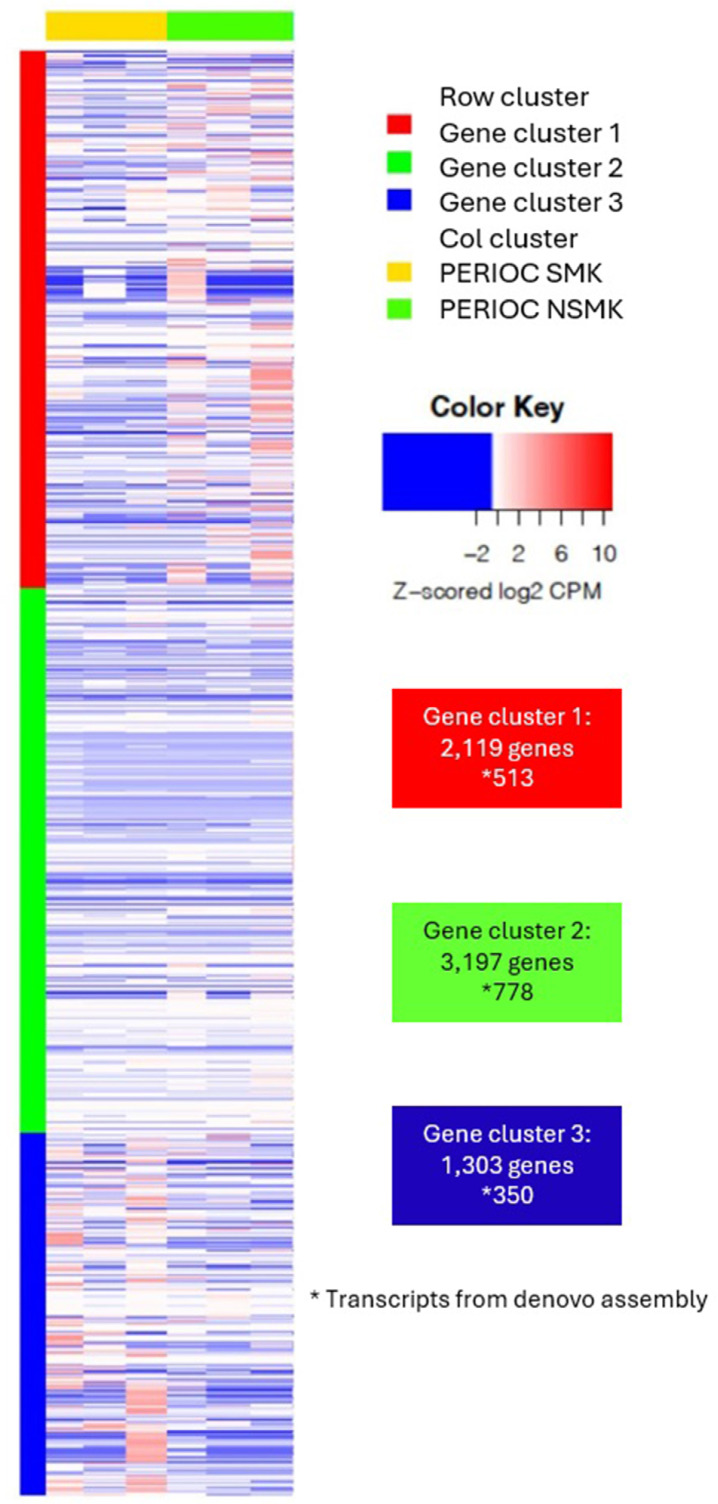
Heatmap analysis of bacterial KEGG orthologues, identified in the metatranscriptomic analysis of subgingival biofilm of the PerioCSmk and PerioCNSmk groups. According to their sequence similarities, the KEGGs were grouped into three different clusters

Figure 1 shows the distribution of the 3351 KEGGs in the subgingival biofilm of both groups (Supplementary Table 1). Differential metatranscriptomic analysis found 304 differentially expressed KEGGs between both groups (PerioCSmk versus PerioCNsmk), 112 overexpressed and 192 underexpressed in the smoker’s group (Supplementary Table 2). KEGGs with positive fold change values indicate overexpression in smokers, whereas negative values, overexpression in non-smokers or underexpression in smokers.

Tables 2 and 3 describe the 10 overexpressed KEGGs with the highest fold change values in each group. Smoking modulated KEGGs related to bacterial metabolism and upregulated genes mainly related to peptidases, inhibitors, and cellular processes. Evaluating the bacterial taxonomy associated with those genes showed that both groups possess genes regulated by members of the red complex of gram-negative pathogens. However, an increase in some KEGGs exclusive of Gram-positive species (e.g., *Actinomyces* genera and *Streptococcus gordonii* species) occurred in the 10 overexpressed KEGGs in PerioCSmk, such as C5a peptidase (K08652), polycystin 1 (K04985), ribonuclease D (K03684), and ABC transport system ATP-binding/permease protein (K21397).

**Table 2:** Top 10 overexpressed genes in PerioCSmk group.

KEGG	Definition	Fold Change	Associated Brites	Associated Taxon
K19956	L-sorbose 1-phosphate reductase	4.05	Carbohydrate metabolism	Interspecies
K04985	polycystin 1	3.99	Signaling and cellular processes	*Actinomyces* sp. oral taxon 448 F0400; *Streptococcus oralis* SK10.
K03762	MFS transporter, MHS family, proline/betaine transporter	3.94	Signaling and cellular processes; Transporters	*Propionibacterium acidifaciens* DSM 21887; *Neisseria* sp. oral taxon 014 F0314; Rothia dentocariosa ATCC 17931; *Actinomyces* sp. oral taxon 448; *Kingella oralis* ATCC 51147.
K08652	C5a peptidase	3.23	Peptidases and inhibitors; Chaperones and folding catalysts	*Streptococcus gordonii Challis* CH1, ATCC 3510; *Eubacterium yurii* subsp. *margaretiae* ATCC 43715; *Streptococcus gordonii Challis* CH1, ATCC 35105.
K01782	3-hydroxyacyl-CoA dehydrogenase / enoyl-CoA hydratase / 3-hydroxybutyryl-CoA epimerase	3.04	Carbohydrate metabolism; Lipid metabolism; Amino acid metabolism; Metabolism of terpenoids and polyketides; and Xenobiotics biodegradation and metabolism	Interspecies; *Pseudoramibacter alactolyticus* ATCC 23263
K22432	caffeyl-CoA reductase-Etf complex subunit CarE	3.02	Unclassified: metabolism	*Tannerella forsythia* ATCC 43037; *Prevotella salivae* DSM 15606
K05520	protease I	2.83	Peptidases and inhibitors	*Prevotella salivae* DSM 15606; *Pseudoramibacter alactolyticus* ATCC 23263; *Actinomyces* sp. oral taxon 448 F0400; *Actinomyces* sp. oral taxon 180 F0310
K03684	ribonuclease D	2.73	Genetic information processing	*Actinomyces* sp. oral taxon 448; *Actinomyces dentalis* DSM 19115; *Actinomyces gerencseriae* DSM 6844; *Actinomyces naeslundii* str. Howell 279; *Rothia mucilaginosa* DY-18
K21397	ABC transport system ATP-binding/permease protein	2.63	Signaling and cellular processes; Transporters	*Actinomyces israelii* DSM 43320; *Actinomyces dentalis* DSM 19115; *Propionibacterium acidifaciens* DSM 21887; *Actinomyces gerencseriae* DSM 6844; *Actinobaculum* sp. oral taxon 183 str. F0552; *Actinobaculum* P1 sp. oral taxon 183 F0552; *Actinomyces* sp. oral taxon 414 strain F0588; *Actinomyces* sp. oral taxon 448 F0400; *Actinomyces* sp. oral taxon 848 F0332
K01365	cathepsin L	2.20	Signaling and cellular processes; Transporters	*Rothia aeria* F0474; *Selenomonas flueggei* ATCC 43531; *Streptococcus gordonii Challis* CH1, ATCC 35105

Casarin RC, Silva RV, Paz HE, Stolf CS, Carvalho LM, Noronha MF, Sallum AW, Monteiro MF

**Table 3:** Top 10 overexpressed genes in non-smokers’ group.

KEGG	Definition	Fold Change	Associated Brites	Associated Taxon
K17500	integrin-linked kinase-associated serine/threonine phosphatase 2C	4.56	Protein families: metabolism	T*annerella* sp. oral taxon BU063; *Tannerella* sp. oral taxon HOT-286
K14437	chromodomain-helicase-DNA-binding protein 7	4.40	Genetic information processing	*Tannerella* sp. oral taxon BU063; *Tannerella* sp. oral taxon HOT-286
K01209	alpha-L-arabinofuranosidase	4.04	Carbohydrate metabolism	*Tannerella* sp. oral taxon BU063; *Prevotella oris* JCM 12252; Prevotella salivae DSM 15606
K17624	endo-alpha-N-acetylgalactosaminidase	3.94	Unclassified: metabolism	*Streptococcus oralis* SK313; *Streptococcus* sp. oral taxon 058; *Streptococcus oralis* SK100; *Streptococcus mitis*; *Streptococcus* sp.
K19181	1,5-anhydro-D-fructose reductase (1,5-anhydro-D-mannitol-forming)	3.82	Unclassified: metabolism	*Selenomonas* sp. oral taxon 138; *Streptococcus oligofermentans* AS 1.3089; *Tannerella* sp. oral taxon BU063; *Tannerella* sp. oral taxon HOT-286; *Prevotella melaninogenica*; *Prevotella* sp. oral taxon 317
K20573	2'-deamino-2'-hydroxyneamine 1-alpha-D-kanosaminyltransferase	3.67	Neomycin, kanamycin, and gentamicin biosynthesis; Transferases	*Tannerella* sp. oral taxon HOT-286; *Alloprevotella tannerae* ATCC 51259; *Prevotella tannerae* ATCC 51259; *Tannerella* sp. oral taxon BU063
K09580	protein disulfide-isomerase A1	3.64	Genetic information processing	*Prevotella*
K14415	tRNA-splicing ligase RtcB (3'-phosphate/5'-hydroxy nucleic acid ligase)	3.54	Genetic information processing	*Desulfobulbus* sp. oral taxon 041; *Prevotella* sp. oral taxon 317; *Corynebacterium matruchotii* ATCC 14266
K18785	beta-1,4-mannooligosaccharide/beta-1,4-mannosyl-N-acetylglucosamine phosphorylase	3.44	Unclassified: metabolism	*Prevotella fusca* JCM 17724; *Abiotrophia defectiva* ATCC 49176; *Prevotella salivae* DSM 15606; *Porphyromonas* sp. oral taxon 279; *Prevotella salivae* F0493; *Prevotella pleuritidis* F0068; Prevotella veroralis F0319; Prevotella *melaninogenica* ATCC 25845; *Capnocytophaga* sp. oral taxon 329; *Prevotella* sp. oral taxon 473; *Prevotella oris* F0302; *Prevotella oulorum* F0390; *Prevotella baroniae* JCM 13447; *Prevotella scopus*; *Capnocytophaga* sp. oral F0381; *Prevotella multiformis*; *Prevotella shahii*; *Prevotella loescheii*; *Prevotella baroniae* F0067
K07796	outer membrane protein, copper/silver efflux system	3.32	Environmental information processing; Signaling and cellular processes; Transporters	*Prevotella loescheii* DSM 19665; *Prevotella melaninogenica* ATCC 25845; *Prevotella histicola* F0411; *Prevotella histicola* JCM 15637; *Prevotella denticola* CRIS 18C-A; *Prevotella* sp. oral F0108; *Neisseria* sp. oral taxon 014 F0314; *Prevotella scopus* JCM 17725; *Prevotella* sp. oral taxon 472 F0295

### Pathways regulated for smokers

Supplementary Table 3 shows the total contribution of KEGGs on several pathways in both groups, whereas Supplementary Table 4, the overall differentially expressed pathways between groups. Smoking significantly modified seven pathways (p<0.05): glycan degradation, galactose metabolism, glycosaminoglycan degradation, oxidative phosphorylation, peptidoglycan biosynthesis, butanoate metabolism, and glycosphingolipid biosynthesis.

**Figure 2:**
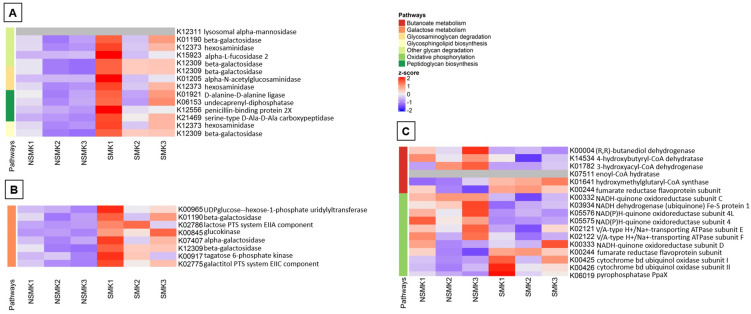
Heatmap analysis of bacterial pathways found by the values (TPM) of each KEGG in patients’ subgingival biofilm. A) Representation of the glycosaminoglycan degradation, glycosphingolipid biosynthesis, other glycan degradation, and peptidoglycan biosynthesis pathways. B) Representation of the galactose metabolism pathway. C) Representation of the butanoate metabolism and oxidative phosphorylation pathways.

Smoking reduced some pathways by downregulating some intrinsic KEGGs (Figure 2). For example, reducing beta-galactosidase (K12309) can negatively affect three different pathways: galactose metabolism, glycosphingolipid biosynthesis, and glycosaminoglycan degradation. In those three pathways, nine, three, and two modules showed downregulation, respectively, which mostly related to carbohydrate metabolism. Similarly, five modules related to enzymes in peptidoglycan biosynthesis were exclusively downregulated, showing that smoking acts in all those pathways only by negative regulation.

Smoking downregulated several genes related to fumarate reductases (i.e., K00244, K00245, K00246, K00247, K00239, K00240, K00241, K00242, K18859, and K18860) and negatively impacted the fumarate participation in butanoate metabolism. Moreover, fumarate reductases constitute one oxidative phosphorylation module, showing downregulation in PerioCSmk.

Moreover, smoking upregulates some KEGGS in the butanoate metabolism pathway, mainly overexpressing KEGGs related to hydroxyacyl-CoA dehydrogenases and hydratases, which can act as oxidoreductases. Oxidative phosphorylation is the most prominent pathway chain regulated by smoking. KEGGs related to NADH dehydrogenases and NADH-quinone oxidoreductases from diverse subunits were upregulated, except for three KEGGs associated with the subunit D, which were downregulated. Smokers showed some alterations in the hydrophilic domain of the mitochondrial matrix membrane and hydron translocation in smokers.

### Antibiotic resistance genes (ARGs)

Smoking altered the levels of antibiotic resistance genes (ARGs) in subgingival biofilm. Using a comprehensive antibiotic resistance database, six genes showed overexpression in PerioCSmk, such as general bacterial porin (which reduces permeability to beta-lactams), the resistance-nodulation-cell division antibiotic efflux pump, and the antibiotic-resistant isoleucyl-tRNA synthetase, most of which are related to *Prevotella sp.* and *Tannerella sp* (Supplementary Table 5).

## Discussion

Current knowledge indicates that the existence of specific pathobionts in biofilm fails to entirely explain how periodontitis occurs. Indeed, an intricate and complex relation between hosts’ response and biofilm, together with the functional profile of the biofilm, play a role in disease development. Moreover, several risk factors could contribute to altering each piece of this puzzle. This study assessed the influence of smoking on the behavior of a dysbiotic subgingival biofilm by a metatranscriptomic approach (whole mRNA sequencing). To our knowledge, this is the first study to investigate the effect of smoking on the functional signature of the microbial community at diseased sites, using the same disease scenario as a control in patients with generalized periodontitis. We found that patients in the smoking group showed significant differences in relation to non-smokers regarding subgingival community gene expression. The main disparities were related to the expression of orthologs associated with metabolism, bacterial-host interactions, and virulence in the subgingival biofilm. These results suggest how a risk factor may distinctly and notably influence disease pathogenesis by altering gene expression in biofilm.

The usefulness of metatranscriptomic data of smoking influence is valuable even with three samples in each group as we used paired-end reads to increase sequencing depth, and a fairly small number of replicates can reach a robust power in data analysis.^7,26^ The results of each sample between groups showed that smoking habits alter the regulation of some pathways, directly influencing subgingival environment dynamics. Smokers’ biofilm was associated with a downregulation of galactose metabolism. The negative regulation of aerobic carbohydrate metabolism genes has been associated with smoke exposure and the establishment of a pathogenic community with increased expression of virulence genes.^6^ Additionally, the alteration in regulatory genes of oxidative phosphorylation in the subgingival environment may favor oxidative stress and the establishment of the anaerobic and reactive oxygen species in the microbial community.^6,24^ The influence of smoking in oral microbiota by oxygen tension alteration and a higher proportion of anaerobic species has offered a striking point associated with smoking in periodontally healthy subjects,^5,28^ pointing out similar mechanisms in dysbiotic environments. Conversely, a study using a predictive tool to determine the metagenomic content found the enrichment of genes related to galactose metabolism and depletion of oxidative phosphorylation pathways in smoker adults affected by periodontitis.^29^ Despite predictive limitations, the authors found an influence of smoking on oral oxygen metabolism, explaining some changes in taxonomic and transcriptome results.

Research has also observed the reduced expression of genes related to fumarate reductase (an enzyme that belongs to anaerobic respiration), which can influence the redox interconversion of fumarate and succinate, altering bacterial development in microaerophilic environments^30^ and modifying the butanoate metabolism. Studies have described butyrate as the main product of gut microbial fermentation, which may exert immunomodulatory effects on intestinal macrophages and maintain intestinal epithelial cells.^31^ In subjects with periodontitis, smoking altering the butanoate chain could explain the more virulent environment and worse clinical conditions. Moreover, the overexpression of genes related to NADH dehydrogenase and oxidative phosphorylation points to the influence of smoking on mitochondrial respiratory systems in the bacterial community, and some studies have shown the functionality of those alterations. It has been shown the role of NADH activity in bacterial cellular metabolism in the virulence induction of *Pseudomonas aeruginosa*, a pathogen associated with chronic infections in human hosts.^32^ The lack of NADH oxidoreductase enzymes in P. aeruginosa may also influence the biofilm formation and production of bacterial toxins,^33^ highlighting the role of NADH-associated genes in diseased environments. The effect of oxidative stress associated with smoking and periodontal disease suffers the influence from changes in the functional profile of the subgingival community, indicating a possible effect on the host immunomodulatory component.

*P. gingivalis* monocultures exposed to tobacco derivatives showed significant functional alterations *in-vitro*,^34^ corroborating our findings. Those metabolites positively regulated proteins involved in virulence, peptide acquisition, oxidative stress, metabolism of fatty acids, coenzymes, and energy production.^35^ The exposure of higher concentrations of tobacco derivatives can also modify the colonization pattern of *P. gingivalis* and favor the invasion of epithelial cells.^34^ By identifying these similarities in our clinical samples, we can more comprehensively depict the expression of this pathogenic community within an ecological context.

The smoking group also showed altered glycan pathways, enriching glycan subproducts. The reduced glycans, glycosaminoglycan degradation, and peptidoglycan biosynthesis in smokers can lead to structural changes in the biofilm. A glycan core on the surface of bacterial cells was associated with modifying and suppressing host immune response by suppressing human Th17 cells.^36^ Besides, the overexpression of immune-related pathways and glycosaminoglycan degradation were related to the asthmatic microbiome, a chronic inflammatory disease of the airways associated with alterations in host immunity.^37^ Thus, the alteration in glycan subproducts may affect host-bacterial interactions, inducing or modulating cellular responses.

In addition to alterations in pathways that can modulate the immune response, a KEGG in the 10 most overexpressed genes in PerioCSmk refers to C5a peptidase, a protease associated with the cell wall of gram-positive bacteria. Its role in hosts’ innate immune response was shown by cleaving and inactivating the C5a peptidase anaphylatoxin C3a and central complement C3 enzyme substrates, which can affect human neutrophil functionality.^38^

Interestingly, the taxonomy data associated with the top 10 transcripts in the subgingival environment showed a major impact of smoking on the *Actinomyces* genera. A recent study using 16S rRNA gene sequencing has shown a significant increase of *Actinomyces* in the saliva of smokers compared to those who never smoked,^28^ suggesting that the phenomenon increases the number of these bacteria and their participation in the gene expression profile. Furthermore, smokers exclusively show top genes regulated by *S. gordonii*. The results of an *in vitro* study showed that nicotine could stimulate the growth and aggregation of planktonic *S. gordonii.*^39^ Together with our results, these data suggest that smoking changes the colonization dynamics of those species and its functional profile in the bacterial community in PerioC-affected patients.

Thus, smoking can modify the gene expression profile of subgingival microbial communities and modify essential pathways. It may also represent a pivotal point to trigger an alteration into the local immune response of periodontal sites. Unsurprisingly, a longitudinal assessment of risk and prognostic factors after periodontal treatment in generalized aggressive periodontitis patients has shown that active smoking is significantly associated with the risk of tooth loss^3^. A recent study targeting the same patient profile confirmed the negative aspect of smoking, observing lower probing depth reduction, CAL gain, and altered balance in pro/anti-inflammatory markers in GCF.^40^ This phenomenon could be related to the aforementioned effects on metabolism and virulence and the overexpression of ARGs in smokers’ biofilm. Increased expression of transcripts related to antimicrobial resistance, such as MerR family transcriptional regulator, outer membrane pore protein F, tellurite resistance protein TerA, also seems to be related to the profile of patients in this study. A recent metagenomic analysis of the subgingival biofilm in periodontal patients has shown the overabundance of genes related to intracellular resistance and multidrug antibiotics efflux pumps in the generalized aggressive periodontitis group.^41^ Thus, smoking also impairs genes related to these functions and may thus negatively impact periodontal treatment outcomes.

In summary, this study examined the additional impact of smoking on young patients with generalized periodontitis and found the overexpression of microbial genes related to host immunomodulation and modifications in metabolic pathways that could influence disease progression. Despite its limited sample size, this study significantly evaluated the number of genes and KEGG pathways. This information is valuable to elaborate hypotheses, validate targeted genes in larger populations, and explore the interplay between these pathways, smoking, and community virulence. This study also emphasizes the importance of studies comparing two disease scenarios to evaluate how biofilm behavior changes, particularly considering the challenges of clinically assessing the direct effects of smoking habits.
